# Microvascular endothelial dysfunction in vascular senescence and disease

**DOI:** 10.3389/fcvm.2025.1505516

**Published:** 2025-02-18

**Authors:** Daniel A. Kasal, Viviane Sena, Grazielle Vilas Bôas Huguenin, Andrea De Lorenzo, Eduardo Tibirica

**Affiliations:** ^1^ Research and Teaching Department, National Institute of Cardiology, Rio de Janeiro, Brazil; ^2^ Internal Medicine Department, State University of Rio de Janeiro, Rio de Janeiro, Brazil; ^3^ Nutrition and Dietetics Department, Fluminense Federal University, Rio de Janeiro, Brazil

**Keywords:** microvascular endothelial dysfunction, inflammation, aging, cardiovascular disease, oxidative stress

## Abstract

Cardiovascular disease (CVD) is the main cause of morbidity and mortality in the adult and the elderly, with increasing prevalence worldwide. A growing body of research has focused on the earliest stage of vascular decline—endothelial dysfunction (ED)—which at the microvascular level can anticipate in decades the diagnosis of CVD. This review aims to provide a prospect of the literature regarding the development of ED as an indissociable feature of the aging of the cardiovascular system, highlighting the role of inflammation in the process. Vascular aging consists of a lifelong continuum, which starts with cell respiration and its inherent production of reactive oxygen species. Molecular imbalance is followed by cellular epigenetic changes, which modulate immune cells, such as macrophage and lymphocyte subtypes. These mechanisms are influenced by lifestyle habits, which affect inflammation hotspots in organism, such as visceral fat and gut microbiota. The process can ultimately lead to an environment committed to the loss of the physiological functions of endothelial cells. In addition, we discuss lifestyle changes targeting the connection between age-related inflammation and vascular dysfunction. Addressing microvascular ED represents a critical endeavor in order to prevent or delay vascular aging and associated diseases.

## Introduction

The increasing proportion of older-age people in modern societies is considered to represent the outcome of long-lasting medical, scientific, and social achievements. However, aging is also typically associated with several changes in the cardiovascular system at different structural and functional levels (1). Cardiovascular disease (CVD) is the main cause of morbidity and mortality in adult and the elderly, with increasing prevalence worldwide (2). Whilst the pathophysiology underlying CVD has been described in detail in the last decades, chronic inflammation has been placed in the center of the molecular and cellular pathways, which ultimately lead to endothelial dysfunction (ED), the first identifiable stage of vascular decline. The mechanisms involved in vascular senescence have been recognized early, acting since the first developmental stages of cellular organisms. During mature life, the physiological processes inherent to growth and homeostasis of organic systems, including cardiovascular, eventually evolve to an unbalanced state, resulting in dysfunction and disease.

The age-related decline in vascular function occurs throughout the vascular tree in humans, ranging from large arteries, such as coronary, to peripheral conduit and resistance vessels, including the microcirculation (3). Accordingly, the vascular response induced by endothelium-dependent vasodilator substances display a progressive decline with age (4, 5). The same deterioration of endothelium-dependent vasodilation has been demonstrated in peripheral conduit vessels including brachial, femoral and popliteal arteries in the elderly (6–9). Predictably, the age-related decline in conduit artery endothelial function is reported to occur at an earlier stage in males than females (6). Moreover, at older age, the cutaneous microvascular endothelial function is also reduced in response to local and systemic physiological or pharmacological stimuli (10–12). Cutaneous microvascular reactivity has been shown to be correlated to microvascular function in different vascular beds, both in terms of intensity and regarding the underlying mechanisms (13).

Vascular aging is considered to be the deterioration in arterial structure and function over time, which ultimately leads to damage of the heart, brain, kidney, and other organs (14–16). Nevertheless, it is important to note that individual vascular age may be very different to their chronological age (17). A number of factors contribute to accelerate vascular aging. Demographic features, such as sex and gender, are well-known important modifiers of cardiovascular system pathophysiology and disease development via genetic, epigenetic, and hormonal pathways (18). Other factors, such as a sedentary lifestyle, poor diet, obesity, and smoking can also hasten vascular aging (14).

In fact, the development of premature vascular aging is influenced by different environmental and genetic factors. Exposure to traditional CVD risk factors, including smoking, obesity, hypertension, diabetes, and hypercholesterolemia promote the development and accumulation of sub-clinical vascular changes that undoubtedly contribute to early vascular aging (14). Genetic factors, represented by DNA damage, also play an important causal role in the dysfunction of endothelial and vascular smooth muscle cells (VSMC) during vascular aging (19, 20), which are associated with structural changes including the increase in vascular diameter and the thickening of arterial wall layers (mainly the intima) in large elastic arteries, throughout the lifespan (21). In this context, epidemiological studies suggest an inverse relationship between DNA integrity and age-related CVD (20). Moreover, extensive evidence supports a central role for DNA damage in the development and progression of macrovascular disease, which in turn support the concept that prolonged exposure to risk factors is a major stimulus for genomic instability within the vasculature (22). Also, epidemiological studies showed that intima-medial thickness of the carotid wall increases 2–3 folds between 20 and 90 years of age (23). Finally, epigenetic factors, including DNA methylation, histone-mediated transcriptional regulation and chromatin remodeling, are also involved in the pathophysiology of vascular aging (16).

In the present review, we define aging by a progressive loss of physiological integrity, leading to impaired function and increased vulnerability to death (24), meaning that aging is the overall process of decline in an organism, which involves the accumulation of senescent cells, among other factors. On the other hand, cellular senescence is characterized by cell-cycle arrest, which prevents the proliferation of damaged cells (25). Thus, while aging and senescence are interrelated, senescence is one of the cellular mechanisms contributing to the broader aging process.

This review aims to provide a prospect of the literature regarding the development of ED as an indissociable feature of the aging of the cardiovascular system, highlighting the role of inflammation in the process. In addition, we sought to provide insights to strategies aimed to slowing down vascular senescence.

### Inflammation, homeostasis and the molecular origins of vascular senescence

Inflammation, while considered as an acute process comprising cellular and molecular phenomena in animals in response to infections or injury (26), is usually described as an advantageous response. On the other hand, a chronic inflammatory “sterile” state, is often view as a pathological process (27).

Systems homeostasis in animals can be defined as a set of mechanisms which preserve physiological processes within an optimal range, enabling maintenance of structure and function, besides the challenges imposed by changes in the environment (28). Accordingly, acute inflammation triggered against a pathogen or tissue damage is adaptive and aimed to restore organic homeostasis on the long term.

A rather dysfunctional, low grade and long-lasting inflammation is recognized in various chronic age-related diseases, including diabetes, hypertension, and CVD (29). At the molecular level, inflammation triggers are classified as pathogen-associated molecular patterns (PAMPs, which are microbial products) and their sterile counterparts, damage-associated molecular patterns (DAMPs, released through tissue damage). These molecules activate pattern recognition receptors (PRR), which are present mainly on innate immune cells, but are also expressed in endothelial cells (30), leading to leukocyte migration through the vessel wall and activation at the lesion site (29). PRR binding on target cells is followed by the production and release of proinflammatory cytokines [interleukin (IL)-1, tumor necrosis factor (TNF)-α], and dysregulation of cell redox equilibrium (31).

Different groups of highly reactive compounds are central to the understanding of the origins of vascular senescence and disease: reactive oxygen species [ROS, such as hydrogen peroxide (H_2_O_2_) and superoxide (O_2_^.−^)], reactive nitrogen species [RNS, including nitric oxide (NO) and peroxynitrite], and the less studied reactive sulfur species (RSS, comprising hydrogen sulfide and persulfides, among others). Theses unstable molecules are involved in the regulation of cell injury, death, cardiovascular pathologies, and inflammation (32, 33).

A key concept in the genesis of sterile chronic immune response is the activation against neoantigens, which are a vast array of molecules derived from healthy host tissues, when exposed to noxious stimuli. The mechanisms which generate neoantigens involve biochemical reactions with ROS. The production of ROS is inherent to physiological processes and immune function, being produced through cellular respiration in mitochondria and also by specific enzymes.

The molecular changes induced by ROS represent epigenetic (for DNA) or post-translational (for proteins) modulation, which are fundamental to physiological gene expression and cell signaling (34). However, depending of the ROS concentration, the cell types involved, the targets and chronicity of the stimulus, the result can be deregulation of homeostasis (35).

One important aspect in the genesis of neoantigens and inflammation is the balance between oxidant and antioxidant producers in vasculature. On the pro-oxidant side, the main enzymes producing ROS are nicotinamide adenine dinucleotide phosphate oxidase (Nox), uncoupled endothelial nitric oxide synthase (eNOS), the mitochondrial respiratory system, and xanthine oxidase (XO) (36). Nox catalyzes the synthesis of superoxide and hydrogen peroxide (37), is involved in cell growth and immune activity, and has been extensively associated with ED. The Nox enzymes (isoforms 1–5), were initially characterized in phagocytes of the innate immune response, and later in VSMCs (38), endothelial cells and adventitial fibroblasts (39, 40). The O_2_^.−^-generating isoforms Nox 1, 2, and 5, are expressed in endothelial and VSMCs, and have been associated with ED, inflammation within the vessel wall, and intimal thickening (41). Nox4 is unique, since it synthetizes H_2_O_2_ and localizes to mitochondria (42).

This set of enzymes has emerged as a primary ROS source in vascular disease and aging. Nox activity is increased in hypertension patients (43) and is upregulated in perivascular tissue upon exposure to cardiovascular risk factors and conditions including smoking (44), diabetes and obesity (45). In a murine model, Nox4 expression in VSMCs increased almost two-fold with aging, which was associated with even higher increase in IL-6 expression (46).

Besides directly generating H_2_O_2_, Nox drives in the uncoupling of eNOS, (which starts producing O_2_^.−^, instead of the vasodilator NO) (47), and converts the antioxidant xanthine dehydrogenase in the O_2_^.−^ and H_2_O_2_ producer XO (48).

On the other hand, important antioxidant mechanisms act to maintain cellular ROS levels under physiological concentrations in cardiovascular tissue, which have been demonstrated to be suppressed in CVD and aging. Special attention, including as a therapeutic target, has been devoted to the transcription factor nuclear factor erythroid 2-related factor 2 (Nfr2). This molecule upregulates a set of proteins with antioxidant actions, such as nicotinamide adenine dinucleotide phosphate quinone oxidoredutase-1, heme oxygenase 1 (HO-1), and glutathione peroxidase, among others (49). HO-1 degrades heme to produce carbon monoxide, capable of suppressing vasoconstriction through increased cyclic guanosine monophosphate (cGMP) (50).

Nfr2 is a downstream target of the renal protein klotho (KL). This hormone synthetized in the kidneys was originally described as an anti-aging molecule. Mice mutant for the KL gene displayed reduced lifespan and a senescent phenotype, including neurological, cutaneous and metabolic derangements. Accordingly, it was named by the original research authors after the Greek mythology deity Klotho, who spins the thread of life (51). The soluble KL protein was shown to attenuate Angiotensin II-mediated apoptosis and senescence in human aortic VSMCs, via activation of Nrf2 and HO-1 (52).

Both Nfr2 and HO-1 induction have been demonstrated to reduce senescence-related oxidative stress in human cell culture studies, in hyperglycemia and smoking conditions, respectively (53, 54). The cardiovascular protective effects through stimulus of the Nfr2 pathway have been studied with the employment of natural products, such as herbal medicines extracts enriched with flavonoids, terpenoids and phenols (55). Besides enzymatic systems reducing oxidative stress, a growing field of study and possible interventions include microRNAs. For instance, miR92-A, has been demonstrated to induce HO-1 and reduce oxidative stress in human endothelial cells (56).

When ROS react with lipids, a heterogeneous group of molecules, known as lipid peroxidation products (LPO) is generated. The degradation of LPO in turn results in reactive aldehydes, which have longer half-life and can diffuse to adjacent tissues (57). LPO-derived aldehydes can react with lipids, proteins and DNA, resulting in vascular pathology.

Both low-density lipopoproteins (LDL) and high-density lipoproteins, (HDL) traditionally recognized as displaying antagonistic actions in atherosclerosis, can react with LPO. The reaction with the former results in oxidized LDL (oxLDL) particles, which display new modified epitopes, capable of triggering macrophage and adaptive immune responses directed to the vessel wall (58).

OxLDL is capable of binding to a specific PRR in endothelial cells, the Toll-like receptor (TLR-4), which activates the nuclear factor kB (NFκB) transcription factor (59). This pathway in turn upregulates a series of proinflammatory mechanisms, including cytokine and mitochondrial ROS production. In addition, NFκB mediates the priming of the inflammasome NLRP3 (which encodes NOD-, LRR- and pyrin domain-containing protein 3). This inflammasome is of particular interest in CVD, since it can be also activated by cholesterol crystals and free fatty acids (60). The end result is the cleavage of pro-IL1β and pro-IL-18 into their mature forms, and the induction of pyroptosis, an inflammatory form of programmed cell death (61). In a murine model, NLRP3 has been demonstrated as upregulated in aging stem cells when compared with cells from young (62), highlighting the molecular link between chronic diseases and aging. The importance of NLRP3 chronic activation in vascular pathophysiology has prompted interest in the pharmacological inhibition of the pathway. Examples of therapeutic compounds with this purpose include glyburide derivatives, colchicine or specific synthetic inhibitors (63). Cytokines downstream from inflammasome activation have also been considered as direct targets in CVD treatment.

The possible use of anti-inflammatory pharmaceuticals for cardiovascular beneficial effects was highlighted by registry studies reporting lower cardiovascular event rates with patients receiving anti-TNF-α pharmaceuticals for autoimmune diseases (e.g., ertanecept or infliximab) (64). This concept was further explored in the first randomized trial with anti-IL1β designed for primary cardiovascular endpoints, showing a reduction in cardiovascular events in patients with previous myocardial infarction (65).

In contrast, HDL oxidation reverses the usual lipoprotein anti-inflammatory properties into proinflammatory, with oxHDL breakdown products being found in higher concentration in atherosclerotic plaques, when compared with serum levels (66). Besides triggering the recognition of host-derived modified molecules, neoantigens have also being implicated in suppressing self-tolerance to host antigens (67), leading to widespread low-grade persistent immune activation and amplification of oxidative stress. In addition to a wide set of modifications in gene expression, protein synthesis and processing, ROS inactivate NO, the main vasodilation mediator, with NO/ROS imbalance being a crucial mechanism to ED and CVD (68).

One important issue and still subject to debate is to determine in which extent these mechanisms are inherent to “healthy” aging or are unique pathophysiological processes. One perspective is that the senescent phenotype and inflammation are epiphenomena which coexist both in aging and age-related diseases, and act synergically, in a vicious cycle (69). Accordingly, the term “Inflammagin” was coined 25 years ago, to refer to the myriad of long-term chronic inflammatory processes, which occur through life (70).

### The role of gut microbiota in vascular dysfunction and aging

The gut microbiota has been recognized as a key regulator of human physiology. The human gastrointestinal tract harbors a vast array of microorganisms (more than 1,000 species in the large intestine) that significantly interact with and affect nutrition, metabolic functions, the immune system, among other increasingly recognized relationships (71). Primarily, the gut microbiota takes part in food digestion and absorption; additionally, in the colon, complex carbohydrates are converted, mainly by bacteria from the *Firmicutes* phylum, into short chain fatty acids such as n-butyrate, acetate, and propionate, which regulate neutrophil function and migration, reduce colonic mucosal permeability, inhibit inflammatory cytokines, and control the redox environment in the cell (72). *Akkermansia muciniphila* is one of the short chain fatty acids producers, and its abundance in the gut is associated with several health benefits in humans. These include maintenance of the integrity of the intestinal barrier and decrease of the occurrence of diabetes, obesity, and inflammatory bowel disease, which are associated with epithelial gut damage and high permeability (73).

Direct and indirect roles of gut microbiota have been described on blood pressure regulation, vascular inflammation and stiffening. Dysbiosis, the change of its composition and function, has been linked to the development and progression of numerous disorders, including CVD (74), and is associated with in an increase of trimethylamine (TMA)-producing bacteria, such as *Escherichia* and *Klebsiella*. TMA is a compound obtained either directly from diet or formed by the metabolism of dietary choline, carnitine, betaine, and ergothioneine by intestinal microflora in the colon (75). TMA is absorbed from the intestine and oxidized to trimethylamine N-oxide (TMAO), in the liver (76). Both observational and experimental studies suggest a positive correlation between increased plasma TMAO concentrations and CVD, such as myocardial infarction, and stroke (77). TMAO was shown to activate NLRP3 in murine endothelial cells, resulting in vascular oxidative stress and endothelial dysfunction (78). This compound has also been associated to the induction of inflammatory markers in mice and in human aortic endothelial cells and VSMCs, through direct activation of the NF-κB signaling cascade, along with promoting the recruitment of activated leukocytes to endothelial cells (79).

On the other hand, the overgrowth of beneficial bacteria (mainly butyrate producers) is associated with lower arterial stiffness. Menni et al. found that gut microbiome diversity was inversely associated with arterial stiffness, evaluated by carotid-femoral pulse wave velocity (PWV). The abundance of *Ruminococcaceae* family bacteria, which are beneficial butyrate-producing bacteria linked to lower endotoxemia, was also associated with lower arterial stiffness (80). In a cross-sectional study, Biruete et al. (81) accessed the relationship between the gut microbiota composition and cardiometabolic risk factors in hemodialysis patients. In their study, bacterial abundance was inversely associated with age. The *Firmicutes/Bacteroidetes* (F/B) ratio was positively associated with traditional risk factors for CVD, such as arterial systolic blood pressure. Also, there was an inverse association between the genus *Faecalibacterium* and carotid-femoral PWV. The authors note that *Faecalibacterium prausnitzii* is a butyrate producer, and that might explain this finding, since there is a known inverse correlation between butyrate production and systemic inflammation (82). Finally, plasma concentration of lipopolysaccharide-binding protein, a marker of bacterial translocation, was negatively associated with butyrate-producing bacteria.

### The modulation of vascular inflammatory response through aging

In the progression of vascular dysfunction which accompanies aging, the interaction between the immune system and vascular tissue plays a key role. A particular subset of CD4+T cells, known as T regulatory cells (Tregs), has received special interest in vascular pathology. This subpopulation of lymphocytes is important to the maintenance of self-antigen tolerance and has anti-inflammatory actions, through the production of transforming growth factor-β and IL-10 (83). Animal studies have shown that the venous infusion of Tregs prevents angiotensin-II and aldosterone-induced hypertension, oxidative stress and preserves microvascular vasodilation (84, 85). A subsequent human study has suggested that Treg populations are downregulated in hypertensive patients (86).

Remarkably, aging has been associated with Treg dysfunction and reduction in tissue and blood concentrations, a process associated with thymus involution (87). Thymic atrophy is also related to the expansion of senescent proinflammatory CD8+ T cells, which develop increased cytotoxic vascular activity (88).

Another important factor leading to increased inflammation through aging is represented by the adipose tissue, currently view as an endocrine organ regulating metabolic diseases and aging through hormones, known as adipokines. These are immunomodulating and metabolically active mediators produced in the adipose tissue, whose functions are not fully understood, but are regulators of local homeostasis as well as systemic inflammation, as they induce systemic elevation of cytokines (89). Chemerin, an adipokine, is associated with obesity, inflammation, angiogenesis, and metabolic syndrome. Adipose tissue expressing low levels of chemerin display a noninflammatory phenotype (90). Calprotectin is another upregulated adipokine in obesity, which is implicated in inflammation via enhancing adhesion of circulating monocytes or recruitment of macrophages (91). On the other hand, adiponectin is an adipokine with anti-inflammatory actions, which reduces adipose tissue inflammation and improves insulin sensitivity (92).

With aging, adipose tissue distribution and gene expression undergo important changes, with an increase in visceral vs. subcutaneous fat, and with the development of proinflammatory and coagulant gene expression. Dysregulated adipokines, such as very low leptin levels, along with increased TNF-α, are associated with subcutaneous lipoatrophy in the elderly and may determine poor prognosis (93). Conversely, upregulated adiponectin and increased insulin sensitivity are associated with longevity, since centenarians reportedly have increased adiponectin levels, which are inversely correlated with body mass index and body fat percentage (93, 94).

### Microvascular endothelial dysfunction (MED) as a precursor of cardiovascular disease

It is currently well-recognized that both microvascular system alterations and subsequent tissue perfusion defects may precede and predict the development of cardiovascular and metabolic diseases, including arterial hypertension, diabetes and metabolic syndrome (95). The microcirculation is a comprehensive term used to describe the terminal vascular network with diameters of less than 300 µm categorized by a network of microvessels including small arteries, arterioles, capillaries, venules, and other cellular components all of which function to meet the oxygen and nutrient requirements of cells, and provide the large surface area needed for blood-tissue exchange (96, 97). Therefore, organs and tissues rely on adequate perfusion by the microcirculation, and suitable microvessel function is essential for the cardiovascular system to achieve this fundamental role (98). Thus, alterations in microvascular function resulting from aging have important consequences for organ function and cardiovascular health (98). Notwithstanding, endothelium-dependent vasodilatation declines with advancing age in humans independently of disease processes (99).

### Early microvascular alterations and aging

Systemic microvascular dysfunction is considered to be a marker of CVD that manifests before the appearance of cardiovascular symptoms, and is a hallmark of the biological aging process (100). In the specific situation of coronary microvascular dysfunction, for instance, a systematic review and meta-analysis showed that coronary microvascular dysfunction is associated with a nearly 4-fold increase in mortality and a 5-fold increase in major adverse cardiac events (101).

The study of low birth weight (LBW) infants offers an insight of how early the determinants of CVD can occur. Diverse mechanisms by which LBW leads to the development of CVD have been proposed, and most involve ED. Several clinical studies have established a link between LBW and impaired endothelial function in the regulation of vascular tone in children and young adults (102). Indeed, birth weight has a significant positive correlation with flow-mediated dilation. There is evidence suggesting an association between LBW and dysfunction in the circulating number and functional capacity of endothelial progenitor cells (103).

Endothelium-derived microparticles (EMPs) are submicron anucleate vesicles that are released in response to apoptotic or activation stimuli. Once in circulation, EMPs bind, fuse and transfer microRNAs to target cells, thus acting as vectors that could mediate several biological processes with favorable or deleterious effects on vascular homeostasis (104). EMPs play an important role in vascular repair, degenerative process modulation, endothelial progenitor cell mobilization and differentiation, acting as crucial indicators of endothelial damage. The link between EMPs and endothelial function in children with LBW remains unclear, although evidence supports that early life events may have a negative impact on the endothelial cell. Children who had lower birth weight showed higher numbers of circulating CD31+/annexin V+ and CD144+ EMPs. In addition, LBW and high levels of both CD31+/annexin V+ and LDL-C were significant risk factors for the presence of microvascular ED (105). A possible mechanistic hypothesis is that changes in the epigenetic signatures occurring during fetal life would lead to an endothelial cell injury and trigger the release of EMPs.

Endothelial function declines with age, since aging is associated with senescence of the endothelium due to increased rate of apoptosis and reduced regenerative capacity (106). Endothelial cell senescence is an age-related process linked to ED, and denotes impairment in vasodilatation or reduction in the production of vasodilators (107). In fact, aging is associated with deficits in endothelial NO availability (108, 109) and NO synthase-dependent vasodilation of arterioles (110). ED in aging is also characterized by a reduced angiogenic capacity and increased expression of adhesion molecules, which amplify the interaction with circulating factors and inflammatory cells (111).

Genetic predisposition to microvascular dysfunction can constitute an important issue of the pathophysiology of cardiovascular diseases, including hypertension, and altered microvascular function might be an essential inherited phenotype in hypertensive patients. In this context, previous studies suggested that microvascular alterations can occur early in the development of hypertension (112). It has also been suggested that factors influencing microvascular growth and anatomy are potential candidate mechanisms in the pathogenesis of hypertension (113). In an elegant study by Noon et al., it has been demonstrated that hypertensive individuals who have hypertensive parents inherit attenuated microvascular function (113). In the offspring of parents with high blood pressure, the maximal skin blood flow response to physiological stimuli and capillary recruitment are blunted in those who have high blood pressure compared with those who have low blood pressure (113). Capillary rarefaction is considered to be a primary microvascular alteration in essential arterial hypertension, and also to be pathognomonic of this disease (114). In this context, it has been demonstrated that infants born at term to mothers with hypertensive disorders of pregnancy have significant structural capillary rarefaction at birth, when compared to term infants born to normotensive mothers (114).

The chronic and progressive increase in blood pressure that occur during aging, as a result of macrocirculatory changes, induce vasoconstriction within the microcirculation that promotes tissue hypoxia and decreases arteriolar and capillary density (115). This phenomena lead to further increase in peripheral vascular resistance, establishing a vicious cycle and finishing in both tissue injury and target organ damage (116) and is equally present in senescence and hypertension (117–119).

It is important to mention that the evaluation of systemic microcirculatory function in human beings is only feasible in the skin or mucous membranes, including the sublingual microcirculation. In fact, the cutaneous microcirculation is considered to be not only an accessible vascular bed but also representative of other organ systems, while allowing the evaluation of mechanisms underlying vascular disease (13). Moreover, it is also noteworthy that not all microvessels are perfused under conditions of normal metabolic demand (120) and that the non-perfused portion of the microcirculation represents a reserve that may be recruited in response to varying metabolic requirements (119). The recruitment of this functional microvascular reserve appears to be more difficult in aged organs, due to impairments in the dynamic control of blood flow (121). The existence of an age-dependent microvascular rarefaction, which may be extended and amplified by hypertension, has already been reported in experimental studies (122). Moreover, this capillary rarefaction can be reversed by long-term effective anti-hypertensive treatment (122, 123), probably resulting in the improvement of target organ damage and slowing the development of hypertension (124).

The reduction of microvascular reactivity during aging can be explained by several mechanisms. For instance, endothelium-dependent vasodilatation pharmacologically induced by acetylcholine - a conventional test for endothelial function - decays with advancing age (99). Moreover, aging is associated with deficits in endothelial NO availability, including the human coronary arterioles (108), and NO synthase-dependent vasodilation of microcirculation, via an increase in superoxide produced by the activation of Nox (110). ROS signaling increases with age, through increased expression of Nox (125), and can impair endothelium-dependent vasodilation (126). The availability of collateral circulation also declines in the aging microvasculature. For example, collateral circulation increase by means of pial anastomoses after middle cerebral artery occlusion is reduced in an aged animal model (127).

Interestingly, vasoconstrictive responses are also altered during aging. In spite of the limitation in dynamic responses, age is associated with chronic vasoconstriction mediated by local activation of Rho-kinase signaling (128). Cutaneous vasoconstriction in response to low body temperature is also impaired in aging, a response that is largely dependent on adrenergic stimulation by norepinephrine (128). The adrenergic hypercontractility associated with aging is homogenous among vascular beds, starting at the same age in all the vascular territories without experiencing further significant impairments as age increases (129). Moreover, during aging, ED is due, at least in part, to the release of endothelium-derived contracting factors (EDCF) that counteract the vasodilator effect of NO. Endothelium-dependent contractions involve the activation of endothelial cyclooxygenases and the release of various prostanoids, which activate smooth muscle thromboxane prostanoid receptors of the underlying vascular smooth muscle (130–132). This type of vascular dysregulation is thought to contribute to additional age-related pathologies including hypertension and erectile dysfunction (133).

### Lifestyle interventions targeting inflammation and vascular dysfunction

Various lifestyle interventions were studied aiming to modulate the inflammation-vasculature axis. Diet-induced changes of the gut microbiota are linked to changes in arterial stiffness. The concept was corroborated by recent studies, in which arterial stiffness was induced in murine models by fecal transplantation from CVD patients (134, 135). In addition, control mice fed with microbiota from obese mice develop changes in gut permeability and increased PWV (136). In a therapeutical experimental study in rats, soy supplementation shifted the cecal microbiota toward a lower F/B ratio and significantly improved aortic stiffness (137).

In humans, cocoa and chocolate, rich in flavonoids and proanthocyanidins, seem to reduce blood pressure levels and cardiovascular risk, with an improvement in measures of vascular health (arterial stiffness and endothelial function), possibly due to the increased production of NO, and antioxidant/anti-inflammatory properties (138). The impact of cocoa flavonoids on vascular health is mediated by metabolites which depend on gut microbiota metabolism (139). An interesting study of acute soy supplementation showed reduced arterial stiffness mediated by equol (a microbial-derived isoflavone metabolite, produced by gut microbiota after soy intake in some individuals of the Western population) (140).

Antibiotic treatment also induces changes in gut microbiota composition, which have been associated with parallel changes in arterial stiffness. In experimental studies, mice treated with antibiotics have displayed reversal of aortic stiffness induced by the Western diet (141). Surprisingly, antibiotic treatment restored arterial stiffness in old mice to normal levels (142). Nonetheless, the role of antibiotics in the modulation of human microbiota for the improvement of cardiovascular health is uncertain. Added to the fact that antibiotics impact the gut microbiota, reducing bacterial diversity and changing relative abundances, it cannot be recommended, at this time, that this class of drugs be used with that purpose (143).

An additional well studied strategy for improving ED and chronic inflammation that accompanies aging is physical activity. The study of Abd et al. compared the impact of six months of aerobic vs. resisted exercise training on inflammatory cytokines and endothelial activation markers among elderly. Both aerobic and resistance exercise training reduced plasma levels of TNF-α, IL-6 and C-reactive protein (CRP), in addition to elevation of IL-10. These findings suggested that exercise training could modulate systemic inflammation biomarkers in elderly individuals, with more significant changes following aerobic exercise training (144). The same pattern of cytokine modulation was found by Santos et al. who demonstrated that moderate aerobic exercise training for 60 min/day, 3 days/week for six months could improve sleep in elderly, with was associated with anti-inflammatory effects (145).

Hyperglycemia-induced ED and insulin resistance are potent risk factors for CVD, and likely contribute to multiple chronic disease complications associated with aging. Regular physical activity has been recommended as an effective approach, together with medications and dietary control, to improve endothelial function in type 2 diabetes (T2D). Skeletal muscle contraction during physical activity increases local blood flow and cardiac output, which results in increased shear stress on vascular endothelium and increased NO production. The systematic review and metanalysis by Lee et al. support the notion that in T2D patients (average age of 59 years), exercise has beneficial effects on endothelial function, with even better results in low/moderate intensity and aerobic training, when compared to moderate/high intensity and resistance subgroups (146). In a cohort study (average age of 40 years), Hong et al. showed that cardiorespiratory fitness is associated with lower levels of TNF-α and IL-1β. These findings were unrelated to body mass index, which could in part be mediated by enhancing the ability of immune cells to suppress inflammatory responses via adrenergic receptors (147).

### Conclusions

**Figure 1:**
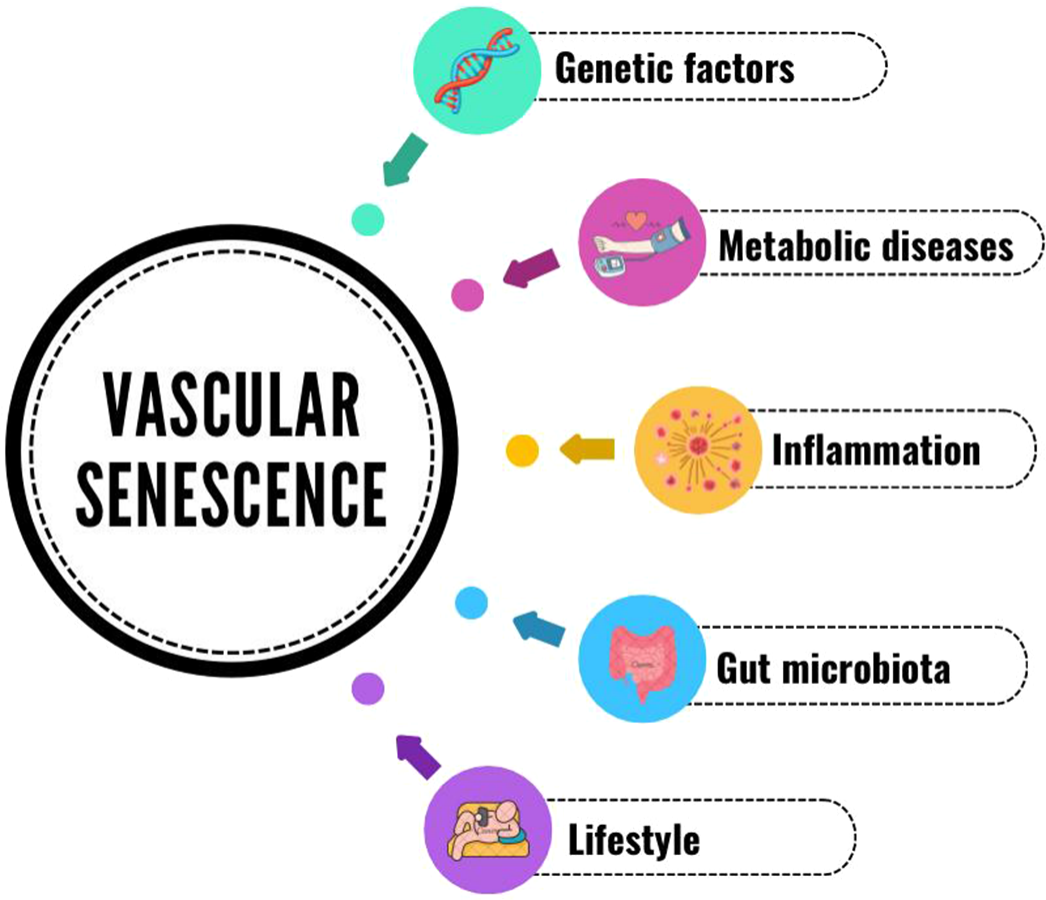
Vascular senescence & disease.

Oxidative stress and inflammation are inherent to vascular aging, act through the lifespan of animals, and are probably set since intrauterine development. Mechanisms essential to homeostasis, depending on the local molecular environment and concentration, can become deleterious and induce ED. Both suppressing proinflammatory and enhancing anti-inflammatory pathways in vasculature are promising targets for treatments aiming to mitigate age-related vascular dysfunction and disease. Gut microbiota is an important milieu where a chronic inflammatory state can convey the progression of vascular decline, but also suitable to interventions through nutritional strategies. Regular physical activity has also been proved in multiple studies to reduce inflammation and ED in both young and the elderly, regardless of the intensity. The vast range of mechanisms coexisting in aging and CVD, and which initially cause ED, is summarized in Figure 1. There is extensive experimental and clinical evidence to support the concept that both microvascular network alterations and tissue perfusion defects may precede and predict the development of cardiovascular and metabolic diseases. Moreover, the functional and subsequent structural alterations in both microvascular reactivity and density, as well as the alterations in the macrocirculation characteristic of physiologic vascular aging, contribute to the development of target end-organ damage (148). Therefore, the microcirculation may be considered an essential target of both the pharmacological and the non-pharmacological treatment of arterial hypertension and other CVD (148).
